# Ophthalmic Artery Doppler Indices at 11–13 Weeks of Gestation in Relation to Early and Late Preeclampsia

**DOI:** 10.3390/jcm14134811

**Published:** 2025-07-07

**Authors:** Nicoleta Gana, Savia Pittokopitou, Filippos Solonos, Alina Perdeica, Marina Fitiri, Kypros H. Nicolaides

**Affiliations:** 1 Harris Birthright Research Centre for Fetal Medicine, King’s College Hospital, London SE5 8BB, UK; gana_nicoleta@yahoo.com (N.G.); savia.pittokopitou@nhs.net (S.P.); filippos.solonos@nhs.net (F.S.); alina.perdeica@nhs.net (A.P.); marina.fitiri@nhs.net (M.F.); 2 Faculty of Medicine, Carol Davila University of Medicine and Pharmacy, 020021 Bucharest, Romania

**Keywords:** preeclampsia, ophthalmic artery doppler, first trimester, prediction, vascular resistance

## Abstract

**Background/Objective:** Preeclampsia (PE) remains a leading cause of maternal and fetal morbidity and mortality. Early prediction is crucial for timely intervention and management. The ophthalmic artery (OA) Doppler assessment in the first trimester has emerged as a potential tool for predicting PE, particularly early PE, with delivery <37 weeks of gestation. This study aimed to evaluate and compare the relationship of ophthalmic artery Doppler parameters at 11–13 weeks of gestation with the subsequent development of early and late PE. **Methods:** A prospective observational analysis was conducted on 4054 pregnant women, including 114 who developed PE. OA Doppler assessment of the pulsatility index (PI) and peak systolic velocity (PSV) ratio, mean arterial pressure (MAP), uterine artery PI (UtA-PI), and serum placental growth factor (PlGF) were compared between women who later developed early PE and late PE with those who did not develop PE. **Results:** In the PE groups, particularly those with early PE, compared to the no PE group, the OA PSV ratio and UtA-PI were higher and PlGF was lower. **Conclusion:** A first-trimester OA Doppler assessment shows promise as a non-invasive method for the prediction of PE. Further prospective, multicenter studies are needed to validate these findings.

## 1. Introduction

Preeclampsia (PE) is a hypertensive disorder of pregnancy characterized by endothelial dysfunction, multi-organ impairment, and an increased risk of maternal–fetal complications [1,2,3,4,5]. The early identification of women at a high risk for PE is essential for initiating preventive strategies, such as aspirin administration before 16 weeks, which has been shown to reduce the incidence of early PE, with delivery < 37 weeks of gestation [6,7].

First-trimester screening for PE uses maternal risk factors, the mean arterial pressure (MAP), the uterine artery pulsatility index (UtA-PI), and maternal serum placental growth factor (PlGF) [8,9]. However, the performance of these markers is not perfect, prompting the investigation of new risk-stratification methods.

Ophthalmic artery (OA) Doppler has been proposed as an additional tool for PE prediction. The OA has a characteristic flow velocity pattern with two systolic peaks: the first corresponds to cardiac ejection, and the second to a reflected wave due to peripheral vascular resistance [10,11]. An increased ratio of these two systolic peaks (PSV2/PSV1 ratio) may indicate increased peripheral vascular resistance, commonly observed in PE [12,13]. Cerebral vasodilation and impaired autoregulation are known features of PE and the OA, the first branch of the internal carotid, provides a non-invasive window into these central adaptations [11,12,13,14]. Unlike uterine artery Doppler [15], OA Doppler assesses systemic cerebrovascular dynamics, offering a complementary pathway to improve risk stratification, especially in low-resource settings where biochemical markers may be inaccessible [16].

In this prospective study, we evaluated the OAPSV ratio and PI in predicting early (delivery < 37 weeks of gestation) and late PE (delivery ≥ 37 weeks) in pregnant women who attended first trimester screening for fetal abnormalities and aneuploidies.

## 2. Materials and Methods

### 2.1. Study Design and Participants

We prospectively recruited 4054 pregnant women with singleton pregnancies who underwent routine first-trimester screening (11^+0^–13^+6^ weeks) at King’s College Hospital, London, UK, between June 2019 and February 2022. In total, 114 women subsequently developed PE. The study was conducted under the Declaration of Helsinki and was approved by the NHS Research Ethics Committee. Participating women provided informed written consent.

We collected the outcomes of pregnancies and divided them into three groups: first those who did not develop PE, second, those who developed early PE, and third, those who developed late PE. The gestational age was established based on a measurement of the crown-rump length (CRL) from the first trimester for spontaneous conception or the embryo transfer date for assisted reproduction techniques (ARTs) [17].

The first trimester visit included a recording of the maternal characteristics and elements of medical history and measurements of MAP, UtA-PI, PlGF, and the OA Doppler PI and OA PSV ratio [8,18,19]. The arterial pressure was measured using a standardized protocol, twice for each arm [18,19], the PI for both uterine arteries was measured transabdominally, and the average of both arteries was recorded [20]. Serum PlGF was analyzed using a biochemical analyzer (BRAHMS KRYPTOR compact PLUS, Thermo Fisher Scientific, Hennigsdorf, Germany). The Doppler studies of the ophthalmic artery were performed by taking two measurements from each eye [21]. All ultrasound scans were performed by doctors who had obtained the appropriate certificate of competence by the Fetal Medicine Foundation. The ophthalmic artery Doppler assessment was performed by doctors who received proper training in the measurement of it.

The inclusion criteria for this observational study were women with singleton pregnancies who presented for a routine scan at 11^+0^–13^+6^ weeks of gestation and the availability of obstetric outcome data. Pregnancies that ended with the delivery of a malformed newborn or stillborn at ≥24 weeks of gestation were excluded.

Data were collected from the hospital records. The primary outcome was the development of PE, classified as early and late PE.

### 2.2. Ophtalmic Artery Doppler Indices

**Figure 1:**
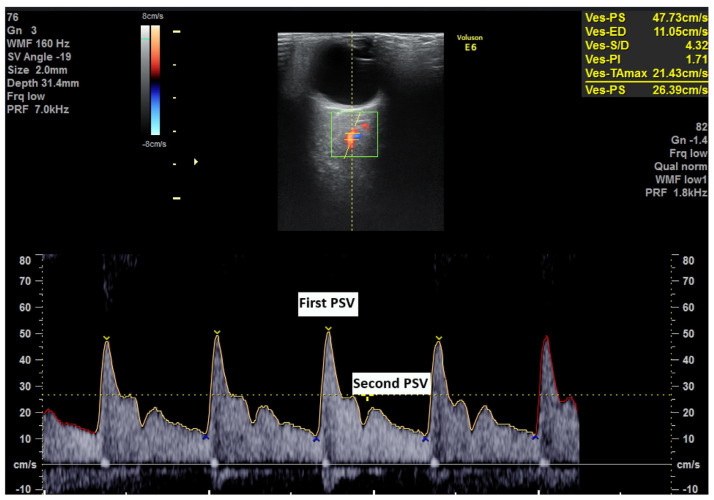
Ophthalmic artery Doppler with indices.

For each patient, we performed four measurements, two for each eye [11]. The OA Doppler is obtained by placing the woman in a supine position. A linear transducer is then placed on the closed upper eyelid. Superior and medially to the optic nerve, color flow Doppler identifies the OA [22]. We recorded 3–5 waves with pulsed wave Doppler (Figure 1). The time used for these four examinations was as short as possible, and the mechanical index was 0.4 [23,24,25]. The OA waveform contains two peak systolic velocities (PSV1 and PSV2). We recorded the ratio between PSV2 to PSV1 and PI and the average of all four measurements [26].

### 2.3. Pregnancy Outcome

PE was defined as new-onset hypertension (systolic blood pressure of ≥140 mmHg or diastolic blood pressure of ≥90 mmHg on two occasions 4 h apart, which appears after 20 weeks of gestation) or chronic hypertension and at least one of the following: proteinuria (≥300 mg/24 h or protein-to-creatinine (PCR) ratio ≥ 30 mg/mmol), renal insufficiency (serum creatinine > 97 μmol/L in the absence of primary renal disease), hepatic dysfunction (serum transaminases more than twice the upper limit of normal), thrombocytopenia (platelet count < 100,000/μL), neurological complications (e.g., cerebral or visual symptoms), or pulmonary edema [26].

### 2.4. Data Analysis

Statistical analysis was performed using Microsoft Excel and IBM SPSS Statistics for Windows, version 30.0 (IBM Corp., Armonk, NY, USA). Kolmogorov–Smirnov and Shapiro–Wilk tests were used to test the normality of the data distribution. MAP, UtA-PI, and PLGF were expressed as multiples of the median (MoM) adjusted to the maternal characteristics [20]. Continuous variables, such as the maternal age, height, weight, BMI, UtA-PI MoM, MAP MoM, PLGF MoM, gestational age at delivery, and interval from last birth, are presented as the mean ± standard deviation (SD).

Comparisons between each of the PE groups and non-PE controls were performed using an Independent Samples *t*-test. Categorical variables, such as smoking, ethnicity, method of conception, previous history of PE, family history of PE, and low-dose aspirin use are expressed as frequencies (*n*) and percentages (%). Statistical comparisons between groups were conducted using a Chi-square test (χ^2^) and one-way ANOVA test. Receiver Operating Characteristic (ROC) curve analysis was performed to evaluate the discriminatory ability of individual variables in differentiating between the early PE and no PE groups and between the late PE and no PE groups. The area under the curve (AUC) was used as a measure of diagnostic performance. A binary logistic regression analysis was performed to evaluate the independent association between OA Doppler parameters and the presence of early and late PE, adjusting for UtA-PI MoM, PlGF MoM, and MAP MoM. For each predictor, odds ratios (ORs) with 95% confidence intervals (CIs) were calculated. Statistical significance was defined by a *p*-value <0.05 for the differences between groups. Since some markers (PlGF and OA PI) had an inverse relationship with the presence of PE, they were transformed (1/x) to allow an interpretation of the ROC curves and regression models.

## 3. Results

The study population of 4054 women included 114 who developed PE (25 of early PE and 89 of late PE). Table 1 presents the maternal characteristics of our study population and the comparison between the groups. The BMI, ethnicity, history of previous PE, UtA-PI MoM, MAP MoM, and family history of PE were significantly different between the groups, but there were no significant differences in maternal age, height, method of conception, smoking, and aspirin use. In the case of OA indices, UtA-PI and MAP values were available for all women in the study population (*n* = 4054), but in the case of PlGF, this was available only in 336 of the cases.

**Figure 2:**
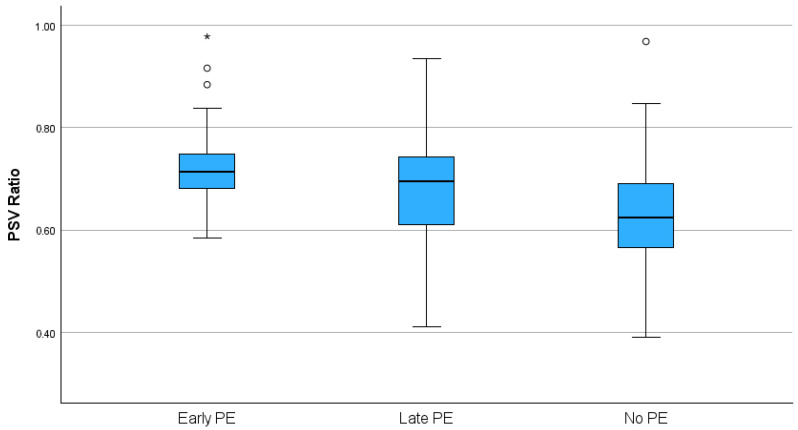
Distribution of the ophthalmic artery PSV ratio (* indicates extreme outlier).

**Figure 3:**
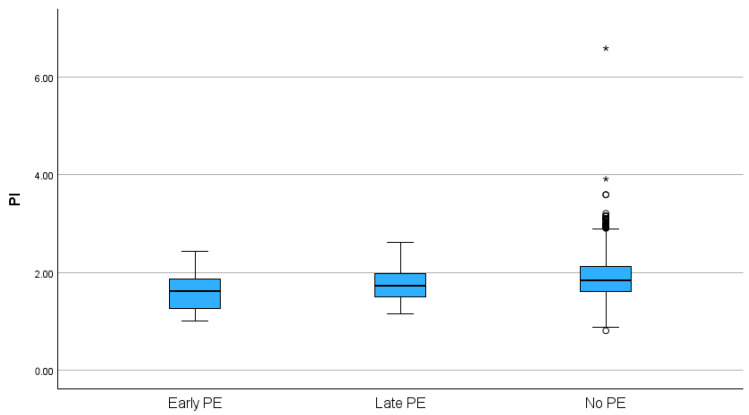
Distribution of ophthalmic artery PI index (* indicates extreme outlier).

Descriptive and comparative statistical analysis: Initially, the mean values of the studied parameters were compared between the groups, using the *t*-test for independent samples. As shown in the box plots in Figure 2 (the box represents the interquartile range, the midline indicates the median value, and the whiskers indicate general variability), the OA PSV ratio was significantly higher in both the early and late PE groups than in the non-PE group (*p* < 0.001). In the case of OA PI, there was no significant difference between the late PE and non-PE groups. In the early PE group, compared to the non-PE group, there was a non-significant trend for a lower OA PI value (Figure 3).

**Figure 4:**
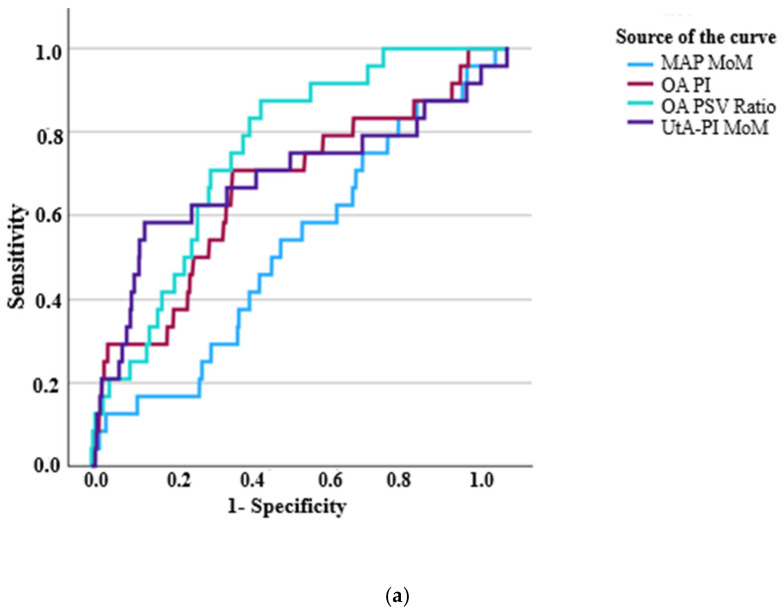

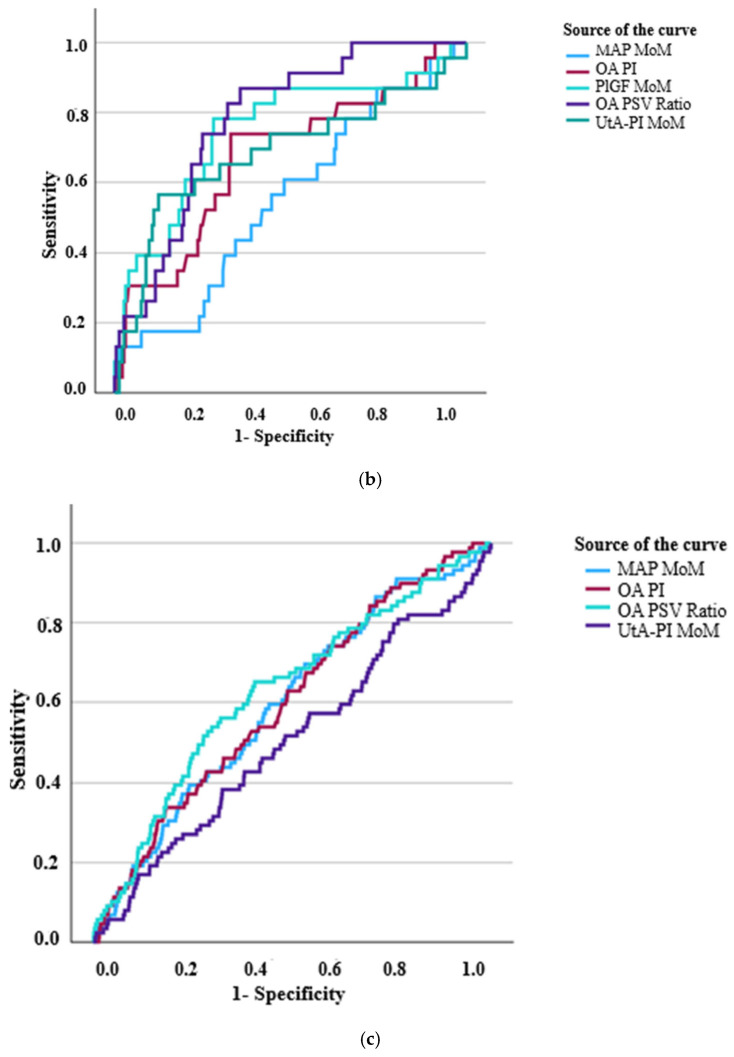

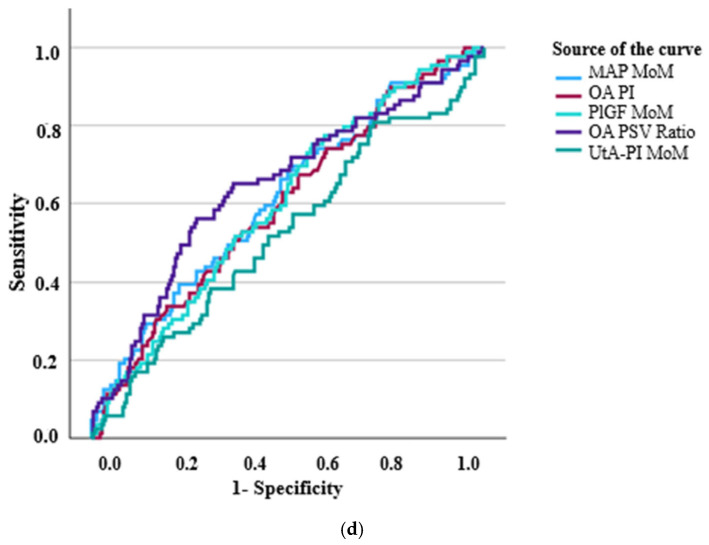
(**a**) ROC curve for OA PSV ratio and PI, UtA-PI MoM, and MAP MoM comparing the early PE with the no PE groups. (**b**) ROC curve for OA PSV ratio and PI, UtA-PI MoM, PlGF MoM, and MAP MoM comparing the early PE with no PE groups (for the subset with PlGF measurements). (**c**) ROC curve for ophthalmic artery PI and PSV ratio, UtA-PI MoM, and MAP MoM comparing the late PE with no PE groups. (**d**) ROC curve for OA PSV ratio and PI, UtA-PI MoM, PlGF MoM, and MAP MoM comparing the late PE with no PE groups (for the subset with PlGF measurements).

ROC analysis: ROC curves (Figure 4a–d) were used to evaluate the performance of OA indices, UtA-PI MoM, MAP MoM, and PlGF MoM in discriminating between women with early PE and late PE from those with no PE.

In the comparison of the early PE group versus the no PE group, there was discriminatory capacity based on the OA PSV ratio, OA PI, UtA-PI MoM, and PlGF MoM (Table 2).

In the cases of the late PE group versus the no PE group, there was discriminatory capacity based on the OA PSV ratio, MAP MoM, and PlGF MoM (Table 2).

Logistic regression analysis: To identify independent predictors of early PE and late PE, binary logistic regression analysis was applied. In the case of early PE versus no PE, independent predictors were the OA PSV ratio (OR = 5576, 95% CI: 21–1,475,892, *p* = 0.002) and UtA-PI MoM (OR = 7.65, 95% CI: 2.62–21.94, *p* < 0.001). Although OA PI was statistically significant based on the *t*-test and ROC analysis, it did not remain an independent predictor in the logistic regression model (*p* = 0.774). In the subset with available PlGF measurements, logistic regression demonstrated the value of this biomarker (OR = 4.16, 95% CI: 1.68–10.29, *p* = 0.002).

In the case of late PE versus no PE, in the binary logistic regression model, there were significant independent contributions from the MAP MoM (OR = 15.96, 95% CI 1.36–187, *p* < 0.05), OA PSV ratio (OR 66.9, 95% CI 3.22–1392, *p* < 0.05), and PlGF MoM (OR 1.8, 95% CI 1.04–3.1, *p* < 0.05).

Table 2 summarize the differences in first-trimester OA Doppler indices and UtA-PI MoM, PlGF MoM, and MAP MoM between pregnancies that later developed early and late PE. The biophysical and biochemical markers exhibited different predictive capabilities depending on the gestational age of developing PE.

In early PE, the OA PSV ratio, UtA-PI MoM, and PlGF MoM demonstrated the best predictive value, whereas for late PE, the OA PSV ratio, MAP MoM, and PlGF MoM were significant independent predictors.

## 4. Discussion

Our study indicates that an OA Doppler assessment in the first trimester may serve as a valuable tool for predicting early PE. Several key insights emerge from our findings.

### 4.1. Hemodynamic Adaptations and Vascular Dysfunction

Early PE (delivery < 37 weeks): The observation of an elevated OA PSV ratio in early PE suggests increased vascular resistance and endothelial dysfunction. This aligns with the findings of Gana et al., who reported that an increased OA PSV ratio at 11–13 weeks of gestation is associated with the subsequent development of preterm PE [21].

Late PE (delivery ≥ 37 weeks): The lower PSV ratio observed in late PE, compared to early PE, may indicate different hemodynamic adaptations in the two conditions. This distinction underscores the heterogeneity of PE pathophysiology, as highlighted in studies emphasizing the need for diverse predictive markers.

### 4.2. Relevance of Ophthalmic Artery Doppler

The OA offers a unique window into cerebral hemodynamics, which can be affected by systemic vascular changes in PE [21]. Unlike UtA-PI, which assesses placental perfusion, OA Doppler evaluates systemic endothelial function. Studies have demonstrated that alterations in OA Doppler waveforms correlate with the development of PE, suggesting its potential as a complementary diagnostic tool [27,28,29,30].

### 4.3. Comparisons with Existing Predictive Models

Traditional first-trimester screening for PE includes the maternal history, MAP, UtA-PI, and serum PlGF. Recent studies have shown that incorporating OA Doppler parameters, particularly the OA PSV ratio, can improve the detection rates of preterm PE when combined with existing biomarkers [21].

### 4.4. Clinical Implications and Future Directions

Integration into Screening Protocols: If validated in larger, multicenter studies, ophthalmic artery Doppler could be integrated into routine first-trimester screening. This integration could enhance early detection and allow for timely interventions.

Personalized Preventive Strategies: Identifying women at a high risk of PE through OA Doppler assessments may facilitate personalized preventive strategies, such as the administration of low-dose aspirin before 16 weeks of gestation, which has been shown to reduce the incidence of early-onset PE [6].

Technological Advancements: Further research should explore the integration of OA Doppler parameters into machine learning algorithms to develop more accurate and individualized risk-prediction models for PE.

### 4.5. Limitations and Next Steps

Study Design: The nature of our study as a single-center setting may limit the ability to generalize the findings. Prospective, multicenter studies with larger sample sizes are necessary to validate the utility of OA Doppler in PE prediction.

Mechanistic Insights: While the association between OA Doppler changes and PE is evident, the underlying mechanisms require further investigation. Understanding whether these vascular changes are a cause or consequence of PE could inform targeted therapeutic approaches.

Standardization of Measurements: The variability in Doppler measurements due to technical factors or operator expertise underscores the need for standardized protocols and training to ensure reproducibility and reliability across different clinical settings.

The small size of the PE groups, especially for early PE (*n* = 25), reduces the statistical power of the tests.

## 5. Conclusions

Our study underscores the potential of a first-trimester OA Doppler assessment as a predictive tool for early PE. Key findings include distinct Doppler patterns associated with early and late PE, highlighting the complexity of the disease and the need for tailored predictive models.

Recent research supports the utility of OA Doppler in PE prediction. For instance, a study demonstrated that an increased OA PSV ratio at 11–13 weeks of gestation is associated with the subsequent development of preterm PE, suggesting that its standalone predictive value is comparable to a uterine artery Doppler evaluation [21]. In this previous study, we found that the OA PSV ratio improves the prediction of PE when combined with other biomarkers, such as MAP, UtA-PI, and PLGF [21].

During pregnancy, an ophthalmic artery Doppler assessment has been shown to predict the subsequent development of PE [31]. These findings suggest that integrating OA Doppler into routine prenatal screening could enhance the early detection and management of PE.

However, further large-scale, prospective studies are warranted to validate these results, explore the underlying mechanisms linking OA Doppler changes to the PE pathophysiology, and establish standardized protocols for its clinical application. Future research should also investigate the cost-effectiveness and feasibility of implementing OA Doppler in diverse healthcare settings to ensure its broad applicability and benefit for maternal and fetal health outcomes.

## Figures and Tables

**Table 1:** Characteristics of the study population.

Characteristics	Early PE (*n* = 25)	Late PE (*n* = 89)	No PE Group (*n* = 3941)	*p*-Value
Maternal age (years)	32.08 ± 6.00	32.39 ± 5.15	32.82 ± 4.82	0.545
Weight (kg)	74.59 ± 17.33	76.61 ± 18.67	69.92 ± 15.04	<0.001
Height (cm)	163.07 ± 8.96	166.81 ± 7.25	165.84 ± 6.77	0.056
BMI (kg/m^2)^	28.17 ± 7.00	27.62 ± 6.46	25.42 ± 5.24	<0.001
Interval from last birth (years)	9.08 ± 8.56	3.68 ± 2.68	3.38 ± 3.01	<0.001
UtA-PI MoM	1.28 ± 0.42	1.03 ± 0.34	1.03 ± 0.31	<0.001
MAP MoM	1.03 ± 0.09	1.04 ± 0.09	1.02 ± 0.08	0.015
Conception				0.591
IVF	2 (8.3%)	6 (6.7%)	176 (4.5%)	
Ovulation drugs	0	1 (1.1%)	18 (0.5%)	
Spontaneous	22 (91.7%)	82 (92.1%)	3851 (95%)	
Ethnicity				<0.001
Black	5 (20.8%)	16 (18%)	510 (12.9%)	
Mixed	5 (20.8%)	7 (7.9%)	133 (3.4%)	
South Asian	2 (8.3%)	4 (4.5%)	268 (6.8%0	
East Asian	0	0	93 (2.4%)	
White	12 (50%)	62 (69.7%)	2937 (74.5%)	
Smoking				0.289
Yes	1 (4.2%)	0	91 (2.3%)	
No	23 (95.8%)	89 (100%)	3850 (97.7%)	
Low-dose aspirin				0.358
Yes	2 (8.3%)	3 (3.4%)	125 (3.2%)	
No	22 (91.7%)	86 (96.6%)	3816 (96.8%)	
Previous PE				<0.001
Parous, no previous PE	4 (16.7%)	26 (29.2%)	1850 (46.9%)	
Parous, previous PE	7 (29.2%)	5 (5.6%)	77 (2%)	
Nulliparous	13 (54.2%)	58 (65.2%)	2014 (51.1%)	
Family history of PE				0.038
Mother	3 (12.5%)	2 (2.2%)	116 (2.9%)	
Sister	1 (4.2%)	1 (1.1%)	40 (1%)	
No history	20 (83.3%)	86 (96.6%)	3785 (96%)	

PE, preeclampsia; BMI, body mass index; UtA-PI, uterine artery pulsatility index; MoM, multiples of median; IVF, in vitro fertilization; MAP, mean arterial pressure; *p*-value, statistically significant < 0.05.

**Table 2:** (a) Comparison of Doppler and biochemical parameters between early preeclampsia and no preeclampsia. (b) Comparison of Doppler and biochemical parameters between late preeclampsia and no preeclampsia.

(a)								
All Patients	Early PE (*n* = 24)	No PE (*n* = 3941)						
Variable	Mean ± SD	Mean ± SD	*p* (*t*-Test)	AUC	*p* (ROC)	OR	OR 95% CI	*p* (Logistics)
1/OA PI	0.64 ± 0.15	0.55 ± 0.15	<0.001	0.671	0.004	1.82	0.029–118	0774
PSV ratio	0.73 ± 0.1	0.64 ± 0.1	<0.001	0.758	<0.001	5576	21–1,475,892	0.002
UtA-PI MoM	1.28 ± 0.42	1.03 ± 0.31	<0.001	0.690	0.005	7.65	2.62–22	<0.001
MAP MoM	1.03 ± 0.09	1.02 ± 0.08	0.233	0.528	0.622	2.67	0.02–330	0.680
**PlGF Subset**	**(*n* = 23)**	**(*n* = 224)**						
1/OA PI	0.64 ± 0.15	0.55 ± 0.11	<0.001	0.674	0.006	0.221	0.001–56	0.590
PSV ratio	0.73 ± 0.96	0.63 ± 0.93	<0.001	0.782	<0.001	958,732	271–3,385,765,152	0.001
UtA-PI MoM	1.25 ± 0.41	1.01 ± 0.31	<0.001	0.685	0.009	8.11	1.72–38	0.008
MAP MoM	1.03 ± 0.085	1.01 ± 0.074	0.204	0.554	0.390	10.5	0.015–7475	0.480
1/PlGF MoM	1.64 ± 0.73	1.08 ± 0.43	<0.001	0.750	<0.001	4.16	1.68–10	0.002
**(b)**								
**All Patients**	**Late PE** **(*n* = 89)**	**No PE** **(*n* = 3941)**						
Variable	**Mean ± SD**	**Mean ± SD**	***p* (*t*-Test)**	**AUC**	***p* (ROC)**	**OR**	**OR 95% CI**	***p*** **(Logistics)**
1/OA PI	0.59 ± 0.12	0.55 ± 0.12	0.002	0.599	0.001	0.80	0.07–9.2	0.860
PSV ratio	0.68 ± 0.11	0.64 ± 0.1	<0.001	0.627	<0.001	66.9	3.22–1392	0.010
UtA-PI MoM	1.03 ± 0.34	1.03 ± 0.31	0.964	0.504	0.895	1.09	0.56–2.1	0.790
MAP MoM	1.04 ± 0.09	1.02 ± 0.08	0.005	0.596	0.001	15.96	1.36–187	0.030
**PlGF Subset**	**(*n* = 89)**	**(*n* = 224)**						
1/OA PI	0.59 ± 0.12	0.56 ± 0.12	0.011	0.596	0.006	0.25	0.009–7.0	0.417
PSV ratio	0.68 ± 0.11	0.63 ± 0.09	<0.001	0.644	<0.001	297	4.9–17,978	0.007
UtA-PI MoM	1.03 ± 0.34	1.01 ± 0.31	0.634	0.523	0.534	1.08	0.48–2.40	0.861
MAP MoM	1.04 ± 0.09	1.01 ± 0.07	0.003	0.609	0.002	32	1.04–1008	0.047
1/PlGF MoM	1.24 ± 0.52	1.08 ± 0.43	0.006	0.600	0.004	1.80	1.04–3.1	0.036

(a) SD—standard deviation; PE—preeclampsia; *p* (*t*-test) = Independent Samples *t*-test; AUC = area under the curve from ROC analysis, representing the variable’s ability to discriminate between early PE and no PE; *p* (ROC) = significance level testing whether AUC differs from 0.5; OR (odds ratio from binary logistic regression); 95% CI = 95% confidence interval for the odds ratio; *p* (logistic) = statistical significance of the predictor in the regression model. (b) SD—standard deviation; PE—preeclampsia; *p* (*t*-test) = Independent Samples *t*-test; AUC = area under the curve from ROC analysis, representing the variable’s ability to discriminate between late PE and no PE; *p* (ROC) = significance level testing whether AUC differs from 0.5; OR (odds ratio from binary logistic regression); 95% CI = 95% confidence interval for the odds ratio; *p* (logistic) = statistical significance of the predictor in the regression model.

## Data Availability

The data presented in this study are available on request from the corresponding author (the data are not publicly available due to privacy and ethical restrictions).

## Abbreviations

The following abbreviations are used in this manuscript:

AUC	Area under the curve
BMI	Body mass index
CI	Confidence interval
IVF	In vitro fertilization
MAP	Mean arterial pressure
MOM	Multiples of median
OA	Ophtalmic artery
OR	Odds ratio
PE	Preeclampsia
PI	Pulsatility index
PlGF	Placental growth factor
PSV	Peak systolic velocity
ROC	Receiver operating characteristic curve
SD	Standard deviation
Ut-A-PI	Uterine artery pulsatility index

## References

[1] Roberts J.M. (2024). Preeclampsia epidemiology(ies) and pathophysiology(ies). Best Pract. Res. Clin. Obstet. Gynaecol..

[2] Sánchez-Aranguren L.C., Prada C.E., Riaño-Medina C.E., Lopez M. (2014). Endothelial dysfunction and preeclampsia: Role of oxidative stress. Front. Physiol..

[3] Nankali A., Malek-Khosravi S., Zangeneh M., Rezaei M., Hemati Z., Kohzadi M. (2013). Maternal complications associated with severe preeclampsia. J. Obstet. Gynaecol. India.

[4] Mikat B., Gellhaus A., Wagner N., Birdir C., Kimmig R., Köninger A. (2012). Early detection of maternal risk for preeclampsia. ISRN Obstet. Gynecol..

[5] Ramos J.G.L., Sass N., Costa S.H.M. (2017). Preeclampsia. Rev. Bras. Ginecol. Obstet..

[6] O’Gorman N., Wright D., Rolnik D.L., Nicolaides K.H., Poon L.C. (2016). Study protocol for the randomised controlled trial: Combined multimarker screening and randomised patient treatment with ASpirin for evidence-based PREeclampsia prevention (ASPRE). BMJ Open.

[7] Konijnenberg A., Stokkers E.W., van der Post J.A., Schaap M.C., Boer K., Bleker O.P., Sturk A. (1997). Extensive platelet activation in preeclampsia compared with normal pregnancy: Enhanced expression of cell adhesion molecules. Am. J. Obstet. Gynecol..

[8] O’Gorman N., Wright D., Syngelaki A., Akolekar R., Wright A., Poon L.C., Nicolaides K.H. (2016). Competing risks model in screening for preeclampsia by maternal factors and biomarkers at 11–13 weeks’ gestation. Am. J. Obstet. Gynecol..

[9] Reddy M., Springhall E.A., Rolnik D.L., da Silva Costa F. (2018). How to perform first trimester combined screening for pre-eclampsia. Australas. J. Ultrasound Med..

[10] Kane S.C., Brennecke S.P., da Silva Costa F. (2017). Ophthalmic artery Doppler analysis: A window into the cerebrovasculature of women with pre-eclampsia. Ultrasound Obstet. Gynecol..

[11] de Oliveira C.A., de Sá R.A.M., Velarde L.G.C., Monteiro V.N.P., Netto H.C. (2012). Doppler Velocimetry of the Ophthalmic Artery. J. Ultrasound Med..

[12] Diniz A.L., Moron A.F., dos Santos M.C., Sass N., Pires C.R., Debs C.L. (2008). Ophthalmic artery Doppler as a measure of severe pre-eclampsia. Int. J. Gynaecol. Obstet..

[13] de Oliveira C.A., de Sá R.A., Velarde L.G., da Silva F.C., doVale F.A., Netto H.C. (2013). Changes in ophthalmic artery Doppler indices in hypertensive disorders during pregnancy. J. Ultrasound Med..

[14] Takata M., Nakatsuka M., Kudo T. (2002). Differential blood flow in uterine, ophthalmic, and brachial arteries of preeclamptic women. Obstet. Gynecol..

[15] Tudor A., Novac L., Camen I.V., Manolea M.M., Sandulescu M.S., Vrabie S.C., Serbanescu M.S., Boldeanu M.V., Istrate-Ofiteru A.M., Dijmarescu A.L. (2023). The Role of Uterine Artery Doppler in the Second and Third Trimesters for Prediction of Preeclampsia and Fetal Growth Restriction Developed as a Consequence of Placental-Mediated Diseases. Curr. Health Sci. J..

[16] de Melo P.F.M.V., Roever L., Mendonça T.M.S., da Silva Costa F., Rolnik D.L., Diniz A.L.D. (2023). Ophthalmic artery Doppler in the complementary diagnosis of preeclampsia: A systematic review and meta-analysis. BMC Pregnancy Childbirth.

[17] Jackson R.A., Gibson K.A., Wu Y.W., Croughan M.S. (2004). Perinatal Outcomes in Singletons Following In Vitro Fertilization: A Meta-Analysis. Obstet. Gynecol..

[18] Wander G.S., McDonagh S.T.J., Rao M.S., Alagesan R., Mohan J.C., Bhagwat A., Pancholia A.K., Viswanathan M., Chopda M.B., Purnanand A. (2022). Clinical relevance of double-arm blood pressure measurement and prevalence of clinically important inter-arm blood pressure differences in Indian primary care. J. Clin. Hypertens..

[19] Pickering T.G., Hall J.E., Appel L.J., Falkner B.E., Graves J., Hill M.N., Jones D.W., Kurtz T., Sheps S.G., Roccella E.J. (2005). Recommendations for blood pressure measurement in humans and experimental animals: Part 1: Blood pressure measurement in humans: A statement for professionals from the Subcommittee of Professional and Public Education of the American Heart Association Council on High Blood Pressure Research. Circulation.

[20] Bhide A., Acharya G., Bilardo C.M., Brezinka C., Cafici D., Hernandez-Andrade E., Kalache K., Kingdom J., Kiserud T., Lee W. (2013). ISUOG Practice Guidelines: Use of Doppler ultrasonography in obstetrics. Ultrasound Obstet. Gynecol..

[21] Gana N., Sarno M., Vieira N., Wright A., Charakida M., Nicolaides K.H. (2022). Ophthalmic artery Doppler at 11–13 weeks’ gestation in prediction of pre-eclampsia. Ultrasound Obstet. Gynecol..

[22] Erickson S.J., Hendrix L.E., Massaro B.M., Harris G.J., Lewandowski M.F., Foley W.D., Lawson T.L. (1989). Color Doppler flow imaging of the normal and abnormal orbit. Radiology.

[23] Flint K., Bottenus N., Bradway D., McNally P., Ellestad S., Trahey G. (2021). An Automated ALARA Method for Ultrasound: An Obstetric Ultrasound Feasibility Study. J. Ultrasound Med..

[24] Varthaliti A., Fasoulakis Z., Lygizos V., Zolota V., Chatziioannou M.I., Daskalaki M.A., Daskalakis G., Antsaklis P. (2024). Safety of Obstetric Ultrasound: Mechanical and Thermal Indexes-A Systematic Review. J. Clin. Med..

[25] Microsoft Word—BMUS Safety Guidelines_2009 Revision_Feb 2010.doc. https://www.bmus.org/static/uploads/resources/BMUS-Safety-Guidelines-2009-revision-FINAL-Nov-2009.pdf.

[26] American College of Obstetricians and Gynecologists (2019). Task Force on Hypertension in Pregnancy. Gestational hypertension and preeclampsia. ACOG Practice Bulletin No. 202. American College of Obstetricians and Gynecologists. Obstet. Gynecol..

[27] Kusuma R.A., Nurdiati D.S., Al Fattah A.N., Danukusumo D., Abdullah S., Sini I. (2023). Ophthalmic artery Doppler for pre-eclampsia prediction at the first trimester: A Bayesian survival-time model. J. Ultrasound.

[28] Saleh M., Naemi M., Aghajanian S., Saleh M., Hessami K., Bakhtiyari M. (2023). Diagnostic value of ophthalmic artery Doppler indices for prediction of preeclampsia at 28–32 weeks of gestation. Int. J. Gynaecol. Obstet..

[29] Gyokova E., Hristova-Atanasova E., Iskrov G. (2024). Preeclampsia Management and Maternal Ophthalmic Artery Doppler Measurements between 19 and 23 Weeks of Gestation. J. Clin. Med..

[30] Gonser M., Vonzun L., Ochsenbein-Kölble N. (2023). Ophthalmic artery Doppler as a marker of pre-eclampsia: Why does it work?. BJOG.

[31] Dai X., Kang L., Ge H. (2023). Doppler parameters of ophthalmic artery in women with preeclampsia: A meta-analysis. J. Clin. Hypertens..

